# Evaluation of the ecological niche model approach in spatial conservation prioritization

**DOI:** 10.1371/journal.pone.0226971

**Published:** 2019-12-20

**Authors:** Fumiko Ishihama, Akio Takenaka, Hiroyuki Yokomizo, Taku Kadoya

**Affiliations:** National Institute for Environmental Studies, Onogawa, Tsukuba, Ibaraki, Japan; Universidade Federal de Goias, BRAZIL

## Abstract

Ecological niche models (ENMs) are widely used in spatial prioritization for biodiversity conservation (e.g. selecting conservation areas). However, it is unclear whether ENMs are always beneficial for such purposes. We quantified the benefit of using ENMs in conservation prioritization, comparing the numbers of species covered by conservation areas selected on the basis of probabilities estimated by ENMs (ENM approach) and those selected on the basis of raw observation data (raw-data approach), while controlling survey range, survey bias, and target size of conservation area. We evaluated three ENM algorithms (GLM, GAM, and random forests). We used virtual community data generated by simulation for the evaluation. ENM approach was effective when survey bias is strong, survey range is narrow, and target size of conservation area is moderate. The percentage of cases in which the ENM approach outperformed the raw-data approach ranged from 0.0 to 33% (GLM), 31% (GAM), and 75% (random forests) depending on conditions. The number of rare species (< 20 presence records) included in the conservation area based on the ENM approach was less than, or the same as, that of the raw-data approach. The unexpectedly limited cases in which the ENM approach was effective in the present research may depend on the conservation target we used (to cover as many species as possible in conservation area). Our results highlight urgent need for evaluating ENM’s effectiveness under other conservation targets for wise use of ENM in conservation prioritization.

## Introduction

Establishment of conservation area networks is central to the conservation of biodiversity, and it is of great consequence to select conservation areas appropriately [1, 2] in the face of the current biodiversity crisis [3]. Sophisticated computational site-selection algorithms have been developed and are widely used to help identify cost-effective conservation area networks that fulfill conservation targets [4]. However, the ability of these algorithms to select appropriately representative conservation areas depends largely on data such as species presence and the extent of vegetation types. Such raw survey data have various limitations, including limited survey ranges, bias in survey ranges, and misclassification of species. A limited survey range and omission errors can reduce the efficacy and options of conservation area networks [5–8], and spatially and taxonomically biased data cause serious problems in conservation prioritization [9, 10]. Without correcting false-positive errors due to misidentification, some species can be excluded from a conservation area even though it was designed to include the species [5].

Considering these limitations of survey data, Margules and Sarkar [1] recommended analyzing or treating raw survey data before using them for conservation prioritization in systematic conservation planning. Using an ecological niche model (ENM) is one of such pretreatments. ENMs generate geographical maps of a species’ probability of presence typically based on correlations between the presence, presence/absence, or abundance of species at multiple locations and the relevant environmental conditions[11, 12]. ENMs are generally expected to improve the comprehensiveness and representativeness of conservation areas by estimating species distributions in unsurveyed ranges and by reducing survey bias [13]. It remains unclear, however, under which condition ENMs can resolve the limitations of survey data for use in spatial prioritization, because they have their own limitations in terms of estimation performance, depending on various conditions. In the case of survey range, for example, it would be difficult to construct an accurate ENM when the survey range is too narrow, and when it is sufficiently large, using an ENM does not improve–and can even worsen–prioritization as compared with using raw data alone. Moreover, prediction by using the ENM approach may have greater uncertainty, and it is not clear how the uncertainty affects the results of conservation prioritization [14].

Thus, the effectiveness of ENMs in conservation prioritization should depend on various conditions. To our knowledge, no research has directly aimed to identify those conditions. Some studies have compared the size and structure of selected conservation networks chosen by using ENMs versus raw survey data [15–18]. These studies used distribution data of real organisms, however, and therefore the true distributions are unknown. Thus, these studies could not refer to the true number of species included in the networks as the baseline for the performance evaluation and could not truly assess whether ENMs actually improved conservation area prioritization. Despite the unclear nature of the benefit of using ENMs and the conditions needed for them to be effective in conservation prioritization, ENMs have been frequently used in studies of conservation prioritization [5, 19–24].

Here, we used realistic distribution data generated by simulating several realistic macro-assemblage properties such as species–abundance relationships. This virtual community data for which we knew the true species distribution allowed us to clarify the conditions under which the use of ENMs resulted in better conservation networks than using raw survey data alone. To evaluate the effectiveness of ENMs, we compared the performance with regard to spatial prioritization between two approaches: (1) the raw-data approach, in which we used only survey data for conservation prioritization; and (2) the ENM approach, in which we estimated ENMs based on the same survey data but used only the predicted probability of species presence for conservation prioritization. We used the “maximal coverage” target as the aim of conservation prioritization [25] in the present study: that is, the size of the conservation area was predetermined (hereafter “target size”), and we selected sites so that they covered as many species as possible. Performance here is defined as the number of species covered in the selected conservation area networks. Though there are variations of “maximal coverage” target to improve persistence of biodiversity in conservation areas networks, we used the simplest conventional target. We considered the effects of the most common limitations of raw survey data, namely survey range and degree of spatial survey bias, on the relative performance of the two approaches.

## Materials and methods

**Fig 1:**
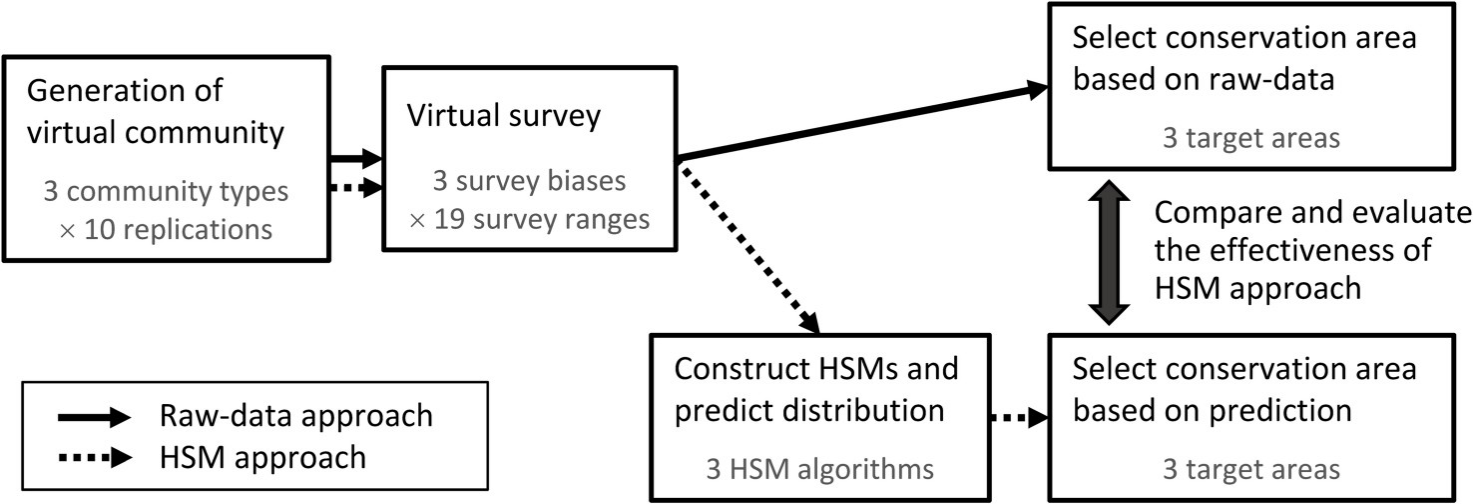
Flow chart of analysis of the effectiveness of the ecological niche model (ENM) approach in conservation prioritization, based on simulated virtual distribution data.

The flow of the analysis is shown in Fig 1, and we explain the details of each analytical process in the following sections.

### Generating virtual community structure

We used artificially generated virtual community data to evaluate the performance of ENMs in conservation prioritization. We generated a species distribution dataset on a one-dimensional space (grid) in which there was one environmental variable, and the environmental value increased linearly as the spatial axis value increased. Both the number of sites (grid cells) and the number of species in the overall range were fixed to 1000.

To generate various and realistic community structures on this environmental space, we developed a program named ComGen (S1 supplemental methods), a generator of an virtual community structure written in Perl. The program generates a dataset of the presence and absence of multiple species at multiple sites that satisfies the three constraints; 1. frequency distributions of number of presence sites per species (rank-abundance curves), 2. frequency distributions of number of species per site (richness patterns), and 3. environmental preferences of individual species. Those assemblage patterns are given to the ComGen program as input.

The rank-abundance curve is expected to affect the accuracy of an ENM, because estimating the distributions of rare species is generally difficult and the curve determines the proportion of rare species in the entire species pool. The species richness pattern among sites influences the effectiveness of a conservation area network, because if many species are concentrated within a small number of sites, many species can be covered by a small conservation area network and vice versa. Therefore, we used realistic rank-abundance patterns: that is, log-linear and log-normal curves and patterns extracted from actual distribution data of various taxonomic groups (amphibians and reptiles, birds, butterflies, dragonflies, freshwater fishes, land and freshwater mollusks, and mammals) in Japan obtained from reports of the Fifth National Survey on the Natural Environment [26]. Log-linear and log-normal curves are commonly used to fit rank-abundance curves of real communities [27]. There are few reports on richness patterns in real communities, so we used log-linear and extracted patterns from the actual distribution data of the Fifth National Survey on the Natural Environment.

We assumed that each species had a unimodal environmental preference. The environmental suitability was a logit conversion of a quadratic function of the environment, and the central value of a suitable environment was chosen randomly. We determined presence/absence of each species considering this habitat suitability and above-mentioned rank-abundance curve and richness pattern.

In the generating process of presence/absence of each species, we preset the number of species at each site and the number of presence sites) of each species so that they meet the above-mentioned richness pattern and rank-abundance curve. The most preferable environmental condition for each species is also preset at random, while the width of the preferable environmental condition depends on the abundance of each species.

Then, we generate a tentative distribution of each species by randomly assigning the predetermined number of presence sites, considering the number of species present at each site, but without considering the environmental preference of the species. Next, we adjust the random tentative distribution to reflect the environmental preference of each species. We randomly select two sites, and a pair of species each of which is present in one of the two sites are swapped, if the swapping makes the distribution more consistent with the environmental preferences. This process is repeated until there is no more swapping to improve consistency with the environmental preferences. The richness pattern and rank-abundance curve are kept unchanged through the swapping process. Details of this process are explained in S1 supplemental methods. Absence sites of each species are defined as the sites where the presence of the species is not assigned.

We generated various community structures changing parameter values of rank-abundance and richness patterns. See S1 Table for the names of parameters we controlled. Because the generation procedure includes stochastic processes and the generated community structure fluctuates randomly, we repeatedly generated 10 communities for each parameter setting. We adopted this iteration number considering the balance between variability of results and calculation time. The calculation time was about 15 min on average per simulation run, including all the processes in Fig 1.

### Virtual survey

We surveyed the virtual community in survey areas of various sizes and with various levels of spatial survey bias. Survey areas were set in blocks, and we controlled the level of spatial sampling bias by changing the number and size of blocks. Survey bias is greater with a smaller number of larger blocks. Although there are other ways to generate spatial survey bias, we chose the block method because the response of ENM effectiveness to survey bias was largest with this method. To set survey grid cells, we first divided the total area (1000 grid cells) into *N* equally sized blocks. When the predetermined proportion of the surveyed range is *R*, 1000×*R*/*N* consecutive grid cells are surveyed in each block. The position of the consecutive survey grid cells in each block is randomly set. When 1000×*R* is indivisible by *N*, the remainder is randomly assigned to survey blocks so that the total number of grid cells surveyed becomes 1000×*R*. We used 19 *R* values from 5% (50 grid cells) to 950% (950 grid cells) at 5% intervals, and three levels of survey bias (no bias, weak bias with *N* = 5 survey blocks, and strong bias with *N* = 2 blocks).

### Ecological niche model

We assumed that species distribution data were presence/absence data, which is one of the most frequently used data type in ENMs[28], and we used three ENM algorithms including two classical statistical algorithms; generalized linear model (GLM)and generalized additive model (GAM), and one machine learning algorithm; random forests (RF) [29]. We chose these algorithms because of the following reasons. GLM and GAM are one of the most popular and established statistical models, and GAM is able to fit non-linear more complicated function than GLM. RF is one of the most popular machine learning algorithms known for good performance and requires relatively short calculation time including tuning parameters for model complexity.

For GLM, we used logit-link function and binomial error distribution, and linear predictor was a quadratic function, which was the same functional form as used in generating the virtual community. Thus, we assumed that we knew the true functional form of each organism’s response to the environmental condition. For GAM, we also used logit-link function and binomial error distribution, and spline curve.

### Selecting the conservation area

We selected the conservation area on the basis of complementarity [30, 31] in both the ENM and raw-data approaches. We used the “maximal coverage” target as the aim of conservation prioritization [25], and we selected sites so that they covered as many species as possible.

Another type of conservation target is the “minimum set” target [25], in which as small a number of sites as possible is selected to cover all species or a certain proportion of species, with no limitation on the size of the conservation area. However, the minimum set target was not applicable in our case because we assumed the survey range was limited and we did not know the true number of species in the whole area.

Target size directly affects the potential number of species to be conserved in the area, and thus it is one of the most important factors that influence the results of conservation prioritization. In the evaluation, we used five target sizes, i.e.1.0, 2.5, 5.0, 9.1, and 17% of the total area. The 9.1 and 17% target size corresponds to the percentage area of national parks in Japan and the Aichi Target of the Convention on Biodiversity, respectively.

### Evaluation of the ENM approach

We evaluated the effectiveness of the ENM approach in conservation area selection based on the following “effectiveness index”: (number of species covered in the conservation area by ENM approach)/(number of species covered in the conservation area by raw-data approach)– 1. An effectiveness index value greater than zero indicates that the ENM approach is beneficial: that is, we could select a conservation area that covers more species by using the ENM approach than by using the raw-data approach.

### Selecting conservation areas by using the raw-data approach

In the raw-data approach, we selected the conservation area on the basis of complementarity by using the greedy algorithm. The greedy algorithm is a heuristic algorithm that uses stepwise analysis to select conservation area [32]. In the first step, the cell with the largest species richness is selected, and then additional cells that add the most additional species are selected step by step.

However, survey range is sometimes insufficient to select a predetermined target size of the conservation area, such as when the survey range is smaller than the target size or when very few species occur in the survey range. Thus, we used the following procedure: (1) we selected a conservation area that covered all the species recorded in the surveyed range, and (2) when the surveyed range was narrower than the target size, we added sites to the conservation area by randomly selecting sites from the unsurveyed range until the conservation area equaled the target size.

### Selecting conservation areas by using the ENM approach

In the ENM approach, we selected the conservation area on the basis of the estimated probability of the presence of each species over the whole area under consideration for conservation. We ignored species that never appeared in the survey range because we could not construct an ENM for such species. We directly used the presence probability and selected conservation areas to maximize the expected number of species represented in the area by the greedy algorithm, as described by Polasky, Camm [33].

There are other ways to select complementary conservation areas on the basis of the probability of species presence, such as converting probability into presence/absence data by using threshold values [16, 34, 35]. We performed a preliminary analysis in which we converted the probability of presence into presence/absence data by using various threshold values, and the results we obtained were qualitatively the same as those with the direct use of probability.

## Results

**Fig 2:**
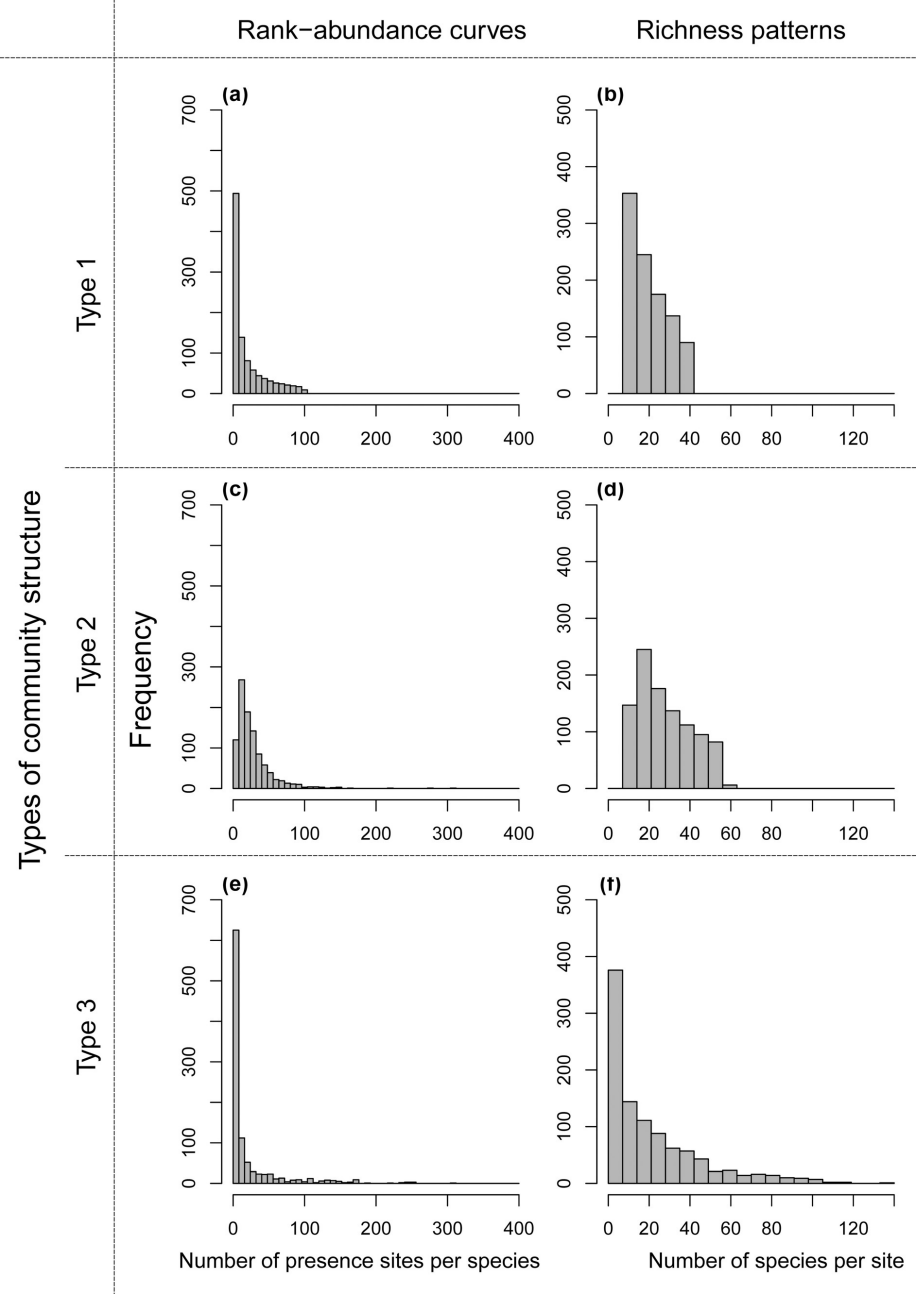
Three combinations of rank-abundance curve and richness pattern used to generate community structure types of virtual distribution data used for the evaluation. (a), (c), (e) Histograms of number of presence sites per species (rank-abundance curve) and (b), (d), (f) number of species per site (richness pattern) in communities with (a), (b) type 1: log-linear, (c), (d) type 2: log-normal, and (e), (f) type 3: realistic patterns of rank-abundance curves in Japanese freshwater mollusks.

Among the various community structures we tried, we identified those cases in which the ENM approach performed best in each type of rank-abundance pattern (i.e., type 1: log-linear, type 2: log-normal, and type 3: realistic patterns). The parameters used to generate these three virtual communities are shown in the S1 Table, and the community structures are illustrated in Fig 2.

For each of these three community structure types, we generated 10 virtual communities. Thus, we performed 25650 runs in total resulting from 3 community structure types × 10 iterations × 19 survey ranges × 3 survey biases × 5 conservation targets × 3 ENMs.

**Fig 3:**
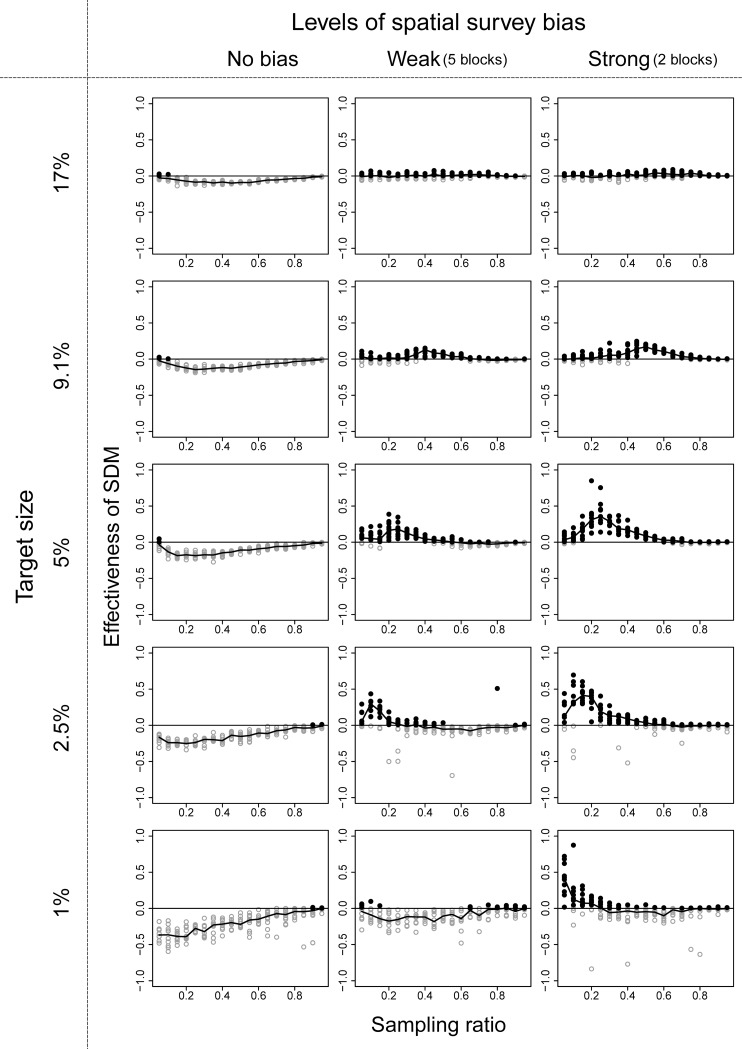
Performance of the ecological niche model (ENM) approach using RF in the community structure type 1 illustrated in Fig 2A and 2B. Black filled circles are values when the ENM approach was beneficial, and gray open circles were the other case. Line chart shows median value in each condition. Target sizes of 9.1% and 17% correspond to the percentage area of national parks in Japan and the Aichi Target of the Convention on Biodiversity, respectively.

**Fig 4:**
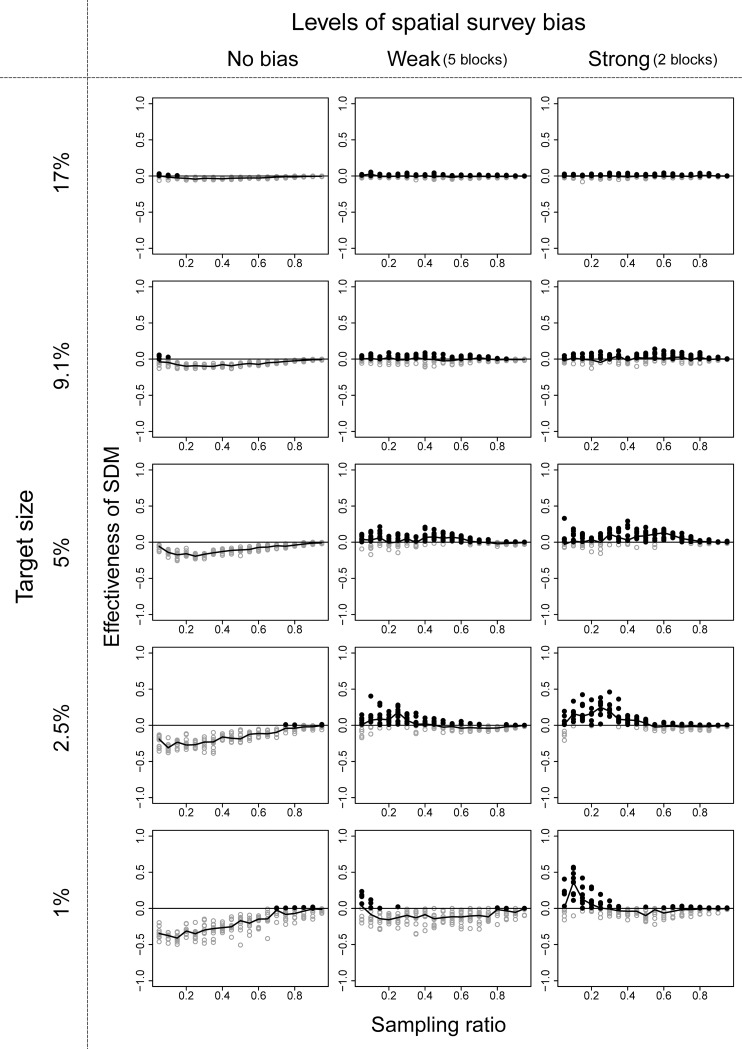
Performance of the ecological niche model (ENM) approach using RF in the community structure type 2 illustrated in Fig 2C and 2D. See Fig 3 for more details.

**Fig 5:**
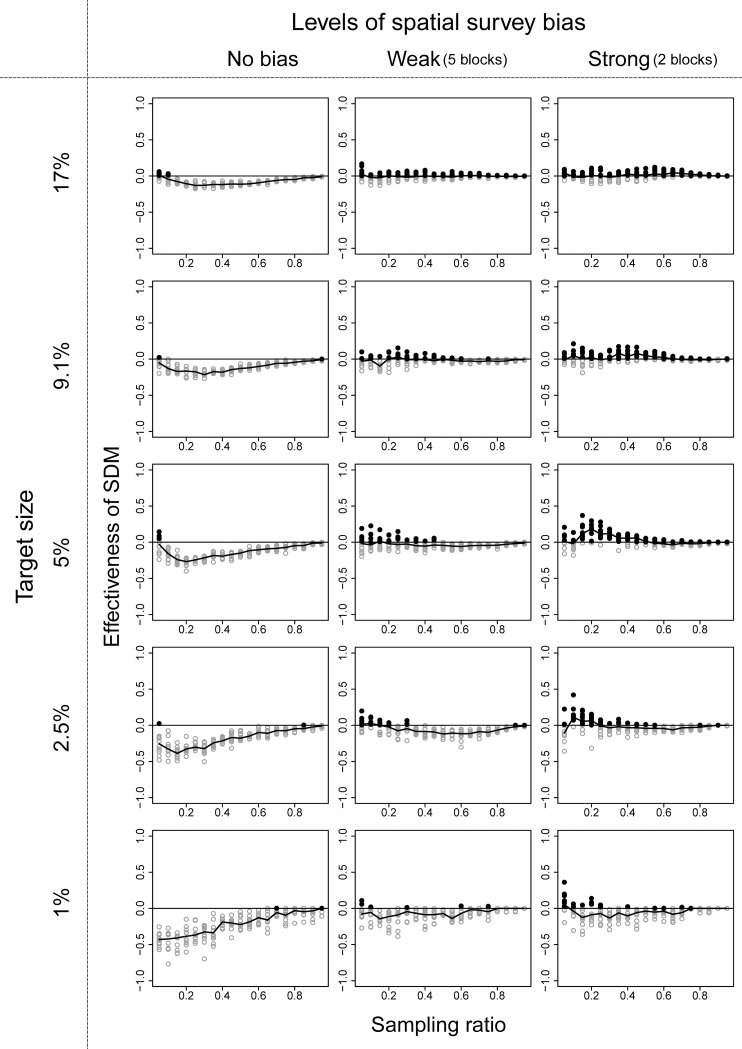
Performance of the ecological niche model (ENM) approach using RF in the community structure type 3 illustrated in Fig 2E and 2F. See Fig 3 for more details.

Among the three ENM algorithms, the performance of RF was the best, and we show the effectiveness of the ENM approach using RF in these three community structures in Figs 3–5. Effectiveness of ENM approach using GLM and GAM are shown in Figs A-F in S1 Figs. The effectiveness of the ENM approach was measured by effectiveness index which takes a value greater than zero when the ENM approach is beneficial. When RF was used, the percentage of cases in which effectiveness index > 0 was 75% (144 of 190 cases) under the best condition (190 cases = 19 sampling ratios × 10 replications with community type 1, target size of 9.1%, and strong survey bias; Fig 3), and it was 29% (2469 of 8550 cases) on average of all conditions we showed in Figs 3–5. When GLM was used, the percentages dropped to 33% (best) and 6.9% (on average), and when GAM was used, the percentages were 31% and 4.5%, respectively.

When we used RF, the conditions ENM approach to be beneficial were that survey bias was large, survey range was narrow to medium, and target size of the conservation area was small to medium. The parameter value of the surveyed range in which the ENM approach became beneficial varied depending on the target size of the conservation area, and the parameter value became smaller with smaller target size. When all three conditions were met, the effectiveness index varied from 0.0 to 1.0. In other conditions, the number of species covered by the selected conservation area based on ENM approach decreased in comparison with the raw-data approach when bias is small and/or target size was small (lower left panels of Figs 3–5). When bias was large and target size was large (upper right panels of Figs 3–5), the numbers of species covered were almost similar (the values of effectiveness index were near 0) between the two approaches.

When we used GAM or GLM, the general tendencies were similar to the results of RF, except that the values of effectiveness index were smaller than 0 even when bias was large and target size was large (upper right panels of S1 Figs and S2 Figs).

**Fig 6:**
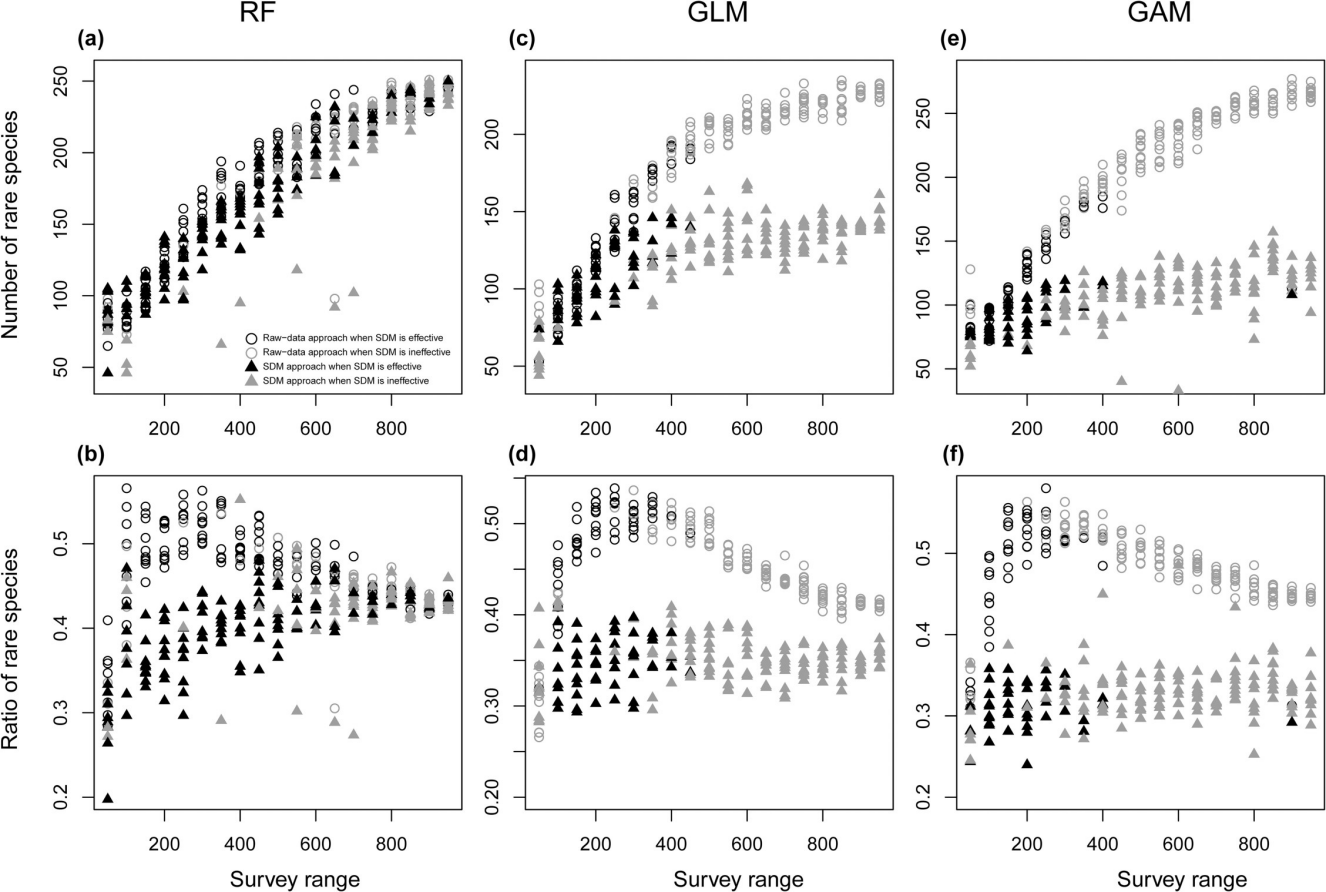
**Ratios of rare species (< 20 presence records) among species in conservation area networks (a), (b), (c), and numbers of rare species covered in conservation area networks (d), (e), (f).** The ecological niche model (ENM) approach using RF is considered effective when the total number of species in the selected conservation area networks is larger than that by using the raw-data approach. The values are in the community structure type 1 illustrated in Fig 2A and 2B, with strong survey bias and a 2.5% target size: that is, in one of the best-performing cases of the ENM approach.

In the conservation areas selected by the ENM approach, the ratio of rare species (species with <20 presence records) covered tended to be smaller than that of the raw-data approach, and this tendency was especially marked when the ENM approach was effective, irrespective of ENM algorithms (Fig 6A–6C). The number of rare species covered by ENM approach using RF tended to be similar to or slightly smaller than that of the raw-data approach (Fig 6D). On the other hand, the number of rare species covered by ENM approach using GLM or GAM was generally smaller than that of the raw-data approach, and this tendency was especially marked when the ENM approach was not effective (Fig 6E and 6f). When the effectiveness of ENM was measured on the basis of the number of rare species by substituting “number of species” in the effectiveness index with “number of rare species,” the parameter ranges for the ENM approach to be beneficial became narrower, and the percentages of cases in which effectiveness index > 0 for rare species using RF, GLM, and GAM were 20, 2.1, and 2.5%, respectively, on average of all conditions for each algorithm (Figs A-I in S3 Figs).

## Discussion

The conditions under which the ENM approach outperformed the raw-data approach in conservation area selection with a target to cover as many species as possible included: (1) large survey bias; (2) narrow to medium survey range; and (3) small to medium target size of conservation area. All of these conditions must be satisfied for the ENM approach to be beneficial from the viewpoint of number of species covered, and the parameter ranges of these conditions were not wide.

### The reason for the conditions under which ENMs to be beneficial in conservation prioritization

Two inherent properties of ENMs are likely to be responsible for the conditions under which ENM-approach to be beneficial in conservation area selection by complementality with the target to cover as many species as possible: (1) ENMs require a certain number of presence records per species for good estimation and cannot properly estimate the distribution of rare species unless the survey range is sufficiently large; and (2) ENMs cannot estimate the distribution range of species that never occur in the survey range.

**Fig 7:**
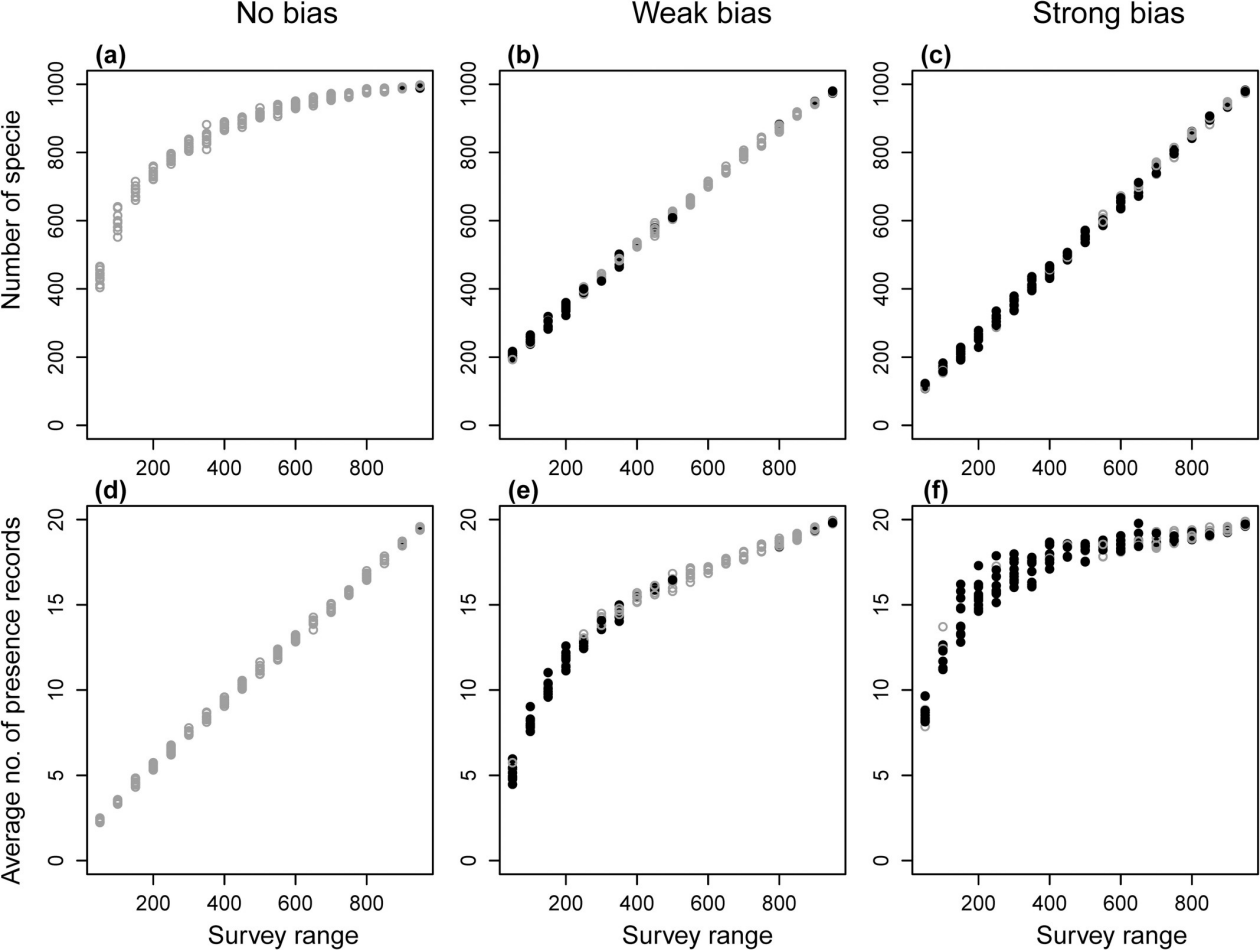
**(a), (b), (c) Numbers of species in surveyed sites and (d), (e), (f) average number of presence records of each species observed at surveyed sites.** With (a), (d) no, (b), (e) moderate (five survey blocks), and (c), (f) strong (two survey blocks) survey bias. The values are in the community structure illustrated in Fig 2A and 2B, with a 2.5% target size. Black filled circles are values when the ENM approach using RF was effective, and gray open circles were the other case.

The parameter conditions under which the ENM approach became beneficial was closely related to the ENMs’ property (1). In the cases of large survey bias (condition 1) and narrow or medium-sized survey range (condition 2), only a few species appeared in the survey range (Fig 7), because only species in limited environmental conditions were sampled under such conditions. There is a trade-off between the total number of species in the survey range and presence records per species, and fewer total species results in increased presence records per species (Fig 7). The trade-off exists because the total number of presence records (i.e., the sum of species richness at all surveyed sites) in survey ranges of the same size is expected to be constant irrespective of their levels of spatial bias, because the species richness at each site was assigned in a spatially random manner in the present simulation (Fig D in S1 Supplemental Methods). Therefore, under conditions 1 and 2, a small number of species frequently appeared in the survey range, and ENMs could be constructed for a larger proportion of the species. In the raw-data approach, however, a reduction in the number of species in the survey range directly resulted in a reduction of conservation efficiency. Thus, under conditions 1 and 2, the ENM approach outperformed the raw-data approach.

When the target size was small to medium (condition 3), the ability of ENMs to predict common species’ distributions was fully utilized. To increase the number of species included in a small conservation area, we had to find sites of high species richness. The species included needed not be rare species, but could be common species, given that we could conserve only a small number of species in a small conservation area. Finding sites where common species are concentrated is exactly the condition under which ENMs work well.

Conditions 2 (narrow to medium survey range) and 3 (small to medium target size) were interrelated. With a smaller target size, the survey range in which a ENM was beneficial became narrower. This is because in a small to medium conservation area, only a few species can potentially be covered, and even a narrow survey range is sufficient to fill such a small conservation area.

The fact that ENMs cannot estimate the distribution range of species that never occur in the survey range is obvious but implies that they have a limited ability to complement spatial information. The main reasons why ENMs were expected to have some benefit in conservation prioritization are that they can complement distribution information in unsurveyed ranges and that they can correct the survey bias. However, a ENM corrects the distribution information of only species that appear in the survey range. The benefit of this correction would be small under the target to cover as many species as possible, because the raw-data approach is also able to cover observed species when the target size is sufficiently large. A more serious problem caused by a biased survey range is that species that live only in unsurveyed range are not considered in conservation prioritization, and an ENM is not able to solve this problem.

Among the three ENM algorithms, RF performed considerably better than GLM and GAM. Though the above-mentioned three conditions for ENM approach to perform better than raw-data approach were common to all three ENM algorithms, RF resulted in negative effectiveness value much less frequently than the other two algorithms especially when target size is large. The superiority of RF is thought to be attributable to its ability to predict distribution of rare species relatively well. The number and ratio of rare species covered in the conservation selected by using RF were notably larger than those of GLM and GAM, especially when survey range was wide (Fig 6), and prediction accuracies were higher when RF was used than when GLM or GAM was used (S4 Fig).

Previous researches compared characteristics of conservation areas selected based on raw-data and ENMs using real distribution data [14–16]. These studies used “minimal area” target, which seeks for minimal conservation area that satisfies a target such as covering all the species. These studies found common characteristics that conservation areas based on ENMs tend to be narrower than that based on raw-data. This is because ENMs estimate wider habitats than known limited presence points, and more frequent overlaps among distribution ranges of different species occur. These studies could not compare effectiveness of raw-data and ENM approaches, because there was no perfect distribution data for the real communities they studied. Our study is the first study which evaluate the conditions for ENM approach to be effective in comparison to raw-data approach using virtual community data as far as we know.

We used ENM for each species, which is the most popular type of distribution models used in conservation prioritization. In contrast, Arponen, et al. [36] compared the efficiency of various combinations of community-level modelling, which model multiple species at once, and maximization algorithms for conservation prioritization by using simulated community data. They showed that maximization of complementary richness–a procedure that accounts for gradients in species richness and non-constant turnover rates of community composition in environmental space–outperforms other approaches including direct selection based on raw data, irrespective of target size they tested. They used virtual community composed of 3000 species in 160 × 160 grid cells with gradient of two environmental variables, biased and fixed narrow survey range (200 sites, randomly scattered within the 25% of total environmental range), and narrow target size (2–64 sites). Direct comparison between their results and our study is difficult because of the difference in the type of modelling, but we can find common point that modelling is effective when survey range is relatively narrow and environmentally biased, and target size is small.

### Implication for future research

It is possible that the unexpectedly narrow parameter ranges in which ENM approach is effective in the present result is dependent on the target of conservation prioritization. We used the “maximal coverage” target to cover as many species as possible. To achieve this target, accuracy of distribution estimation of rare species is important, and this situation is unfavorable to the ENM approach. There are other possible targets for conservation prioritization [25]. For example, when the target is to better cover the distribution range of well-known species in the conservation area, it would be just the case in which ENM-approach is very useful. Although it is beyond the scope of the current study, it is promising avenue for future research to examine relationships between target types and performance of ENMs and to look for the target that has higher affinity with ENM approach.

In addition, the setting in the current analysis to make up for the shortage of conservation area by randomly selecting sites from unsurveyed range when the survey range is insufficient in the raw-data approach may be unrealistic. In reality, we would not select a conservation area in such a way. Another way may be to use a hybrid approach that combines an ENM with raw data. For example, one could select a conservation area on the basis of the raw-data approach and then make up for the shortage by selecting additional sites based on prediction by ENMs. Or, one could select a conservation area on the basis of the raw-data for rare species or species of low ENM accuracy, and ENMs for other species. We performed a preliminary analysis of such a hybrid approach, but among all our attempts there was no case in which the hybrid approach performed better than the raw-data approach. This is probably because an ENM cannot provide any profitable information as compared with random selection of sites owing to its limited ability to complement spatial information (an ENM’s second property). However, there are various possible ways to combine ENMs and raw data, and further analysis of such hybrid approaches may be warranted.

There would be room to improve reality of virtual community structure. We generated virtual communities which satisfy three constraints, i.e. rank-abundance curve, richness pattern, and habitat suitability of each species. We did not consider environmental or spatial structures in richness patterns, but in reality, existence of such structures is well known (e.g. latitudinal gradient in species diversity). In addition, we considered only one environmental variable in this research. Developing algorithm to generate virtual community which satisfy spatio-environmental structures in richness pattern and having multiple environmental variables is challenging, but it would be very interesting to validate the effect of these factors on the performance of ENM approach.

Our present analysis is the first step to understand the characteristics of ENM approach in conservation prioritization, and the present results highlight the importance of future researches in the above-mentioned perspectives for wise use of ENM in conservation prioritization.

## Supporting information

S1 Supplemental Methods Generating virtual community structure by using the ComGen package. (PDF)

S1 Table Settings of the ComGen package used to generate virtual community structures for the analysis. (PDF)

S1 Figs The effectiveness of ENM approach using GLM. (PDF)

S2 Figs The effectiveness of ENM approach using GAM. (PDF)

S3 Figs The effectiveness of ENM approach using Random Forests measured on the basis of the number of rare species in the selected conservation area. (PDF)

S4 Figs Accuracy of each ENM measured by AUC (area under the curve). (PDF)

S1 Data The presence/absence distribution data of each species in the virtual community used for the analysis in Figs 3–5. The data is “list” format of the statistical software R, and the list is composed of four hierarchies (1. the Figure in which the data used, 2. target size, 3. level of the spatial survey bias, and 4. 10 iterations).
(ZIP)
