# Supplementary material for: Evaluation of the ecological niche model approach in spatial conservation prioritization
Source: PLoS One. 2019 Dec 20;14(12):e0226971. doi: 10.1371/journal.pone.0226971 (PMC6924678; doi:10.1371/journal.pone.0226971)
Supplement: S1 Supplemental Methods — (PDF) [file pone.0226971.s001.pdf]

S1 Supplemental Methods. Generating virtual community structure by using the ComGen package

ComGen, a generator of a virtual community structure, is a collection of codes written in Perl. It generates a dataset of the presence and absence of multiple species at multiple sites. The output of ComGen is a two-dimensional matrix, the rows of which represent sites and the columns of which represent species. The elements of the matrix are either 1 (present) or 0 (absent) (Fig A).

	Species 1	Species 2	Species 3	Species 4	…
Site 1	0	1	0	0	
Site 2	1	1	0	0	
Site 3	0	1	0	0	
Site 4	1	1	0	1	
Site 5	0	1	1	0	
Site 6	0	0	0	0	
Site 7	1	1	0	0	
Site 8	1	0	0	1	
Site 9	0	0	1	0	
Site 10	0	0	0	1	
…					

Fig A

The parameters controlling the pattern of the number of present sites among species, the species richness pattern among sites, and the environmental preferences of individual species can be manipulated. Before generating the dataset using ComGen, parameter values are set in sequence as follows, because these parameters are interrelated.

First, the number of sites is set. Sites are assumed to be arranged uniformly along a hypothetical environmental gradient, such as temperature.

Second, the total number of species and number of presence sites (N_p) of individual species are set. By default, the plot of N_p of each species against the rank order of N_p of each species (i.e., a rank-abundance curve) is assumed to follow a log-linear pattern (Fig B). The variation of N_p among species is controlled by manipulating the slope of the log-linear relationship. If necessary, the N_p of individual species can be chosen as desired.

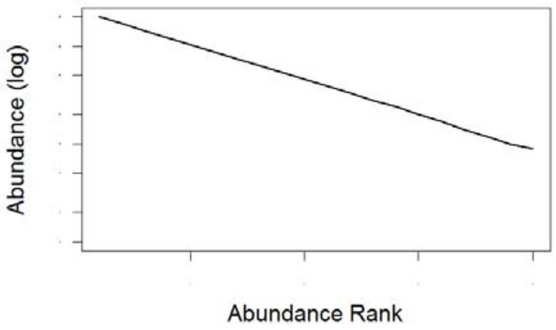

Fig B

Third, species richness (i.e., the number of species) at each site is determined. The species richness pattern among sites is assumed to follow a log-linear pattern (Fig C). The mean richness is adjusted so that the sum of the species richness over all the sites is equal to the sum of the N_p values of all species. The variation of species richness among sites is controlled by manipulating the slope of the log-linear relationship. Then the determined species richness is assigned to individual sites randomly, irrespective of the environmental conditions of the sites.

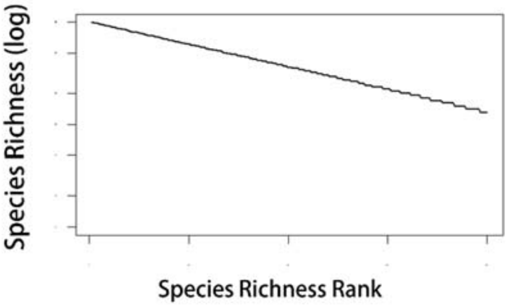

Fig C

Fourth, habitat preference (i.e., the range of suitable environments) is determined for each species. The centers of the habitats of all species are located randomly over the entire environmental gradient (Fig D). The width of the habitat preference of each species depends on the number of presence sites of the species. More abundant species have wider habitat preferences.

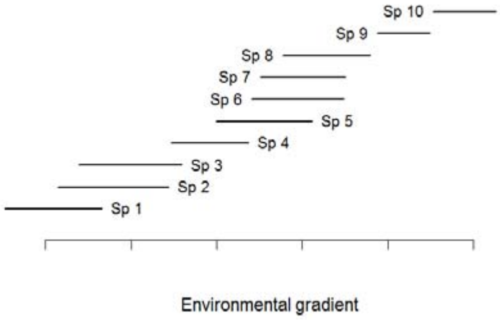

Fig D

Once the parameters are set, ComGen generates a presence/absence dataset. It first generates a tentative distribution of each species by randomly assigning the predetermined number of presence sites, considering the number of species present at each site, but without considering the environmental preference of the species. Next, we adjust the random tentative

distribution to reflect the environmental preference of each species. We randomly select two sites, and a pair of species each of which is present in one of the two sites are swapped, if the swapping makes the distribution more consistent with the environmental preferences. This process is repeated until there is no more swapping to improve consistency with the environmental preferences. The richness pattern and rank-abundance curve are kept unchanged through the swapping process. Absence sites of each species are defined as the sites where the presence of the species is not assigned.

The distribution of each species is not necessarily confined to its potential habitat after the adjustment. There may be no solution satisfying all requirements if the values of parameters are incompatible. Thus, the generated dataset was checked for consistency with the environmental preference of each species by examining the ratio of presence data outside habitats and graphic expression of the distribution of individual species before conducting a conservation area selection analysis. There was no case in which the environmental preference of a species was violated in the parameter range we used in the analysis.
