# Supplementary material for: Evaluation of the ecological niche model approach in spatial conservation prioritization
Source: PLoS One. 2019 Dec 20;14(12):e0226971. doi: 10.1371/journal.pone.0226971 (PMC6924678; doi:10.1371/journal.pone.0226971)
Supplement: S1 Table — (PDF) [file pone.0226971.s002.pdf]

S1_Table Settings of the ComGen package used to generate virtual community structures for the analysis

Setting ID	Community type 1 (Figs 2a,b and Fig. 3)	Community type 2 (Fig2 c,d and Fig. 4)	Community type 3 (Figs 2e,f and Fig. 5)
**Number of grid cells**	1000	1000	1000
**Number of species**	1000	1000	1000
**Type of rank-abundance pattern**	log-linear (maximal abundance = 100, slope = 0.005)	log-normal (mean = 3.0, SD = 0.8)	extracted from actual distribution data (freshwater mollusks)
**Type of richness pattern**	log-linear (slope determined such that the ratio of maximum and minimum richness was 2)	log-linear (slope was determined such that the ratio of maximum and minimum richness was 2)	extracted from actual distribution data (freshwater mollusks)
