# Supplementary material for: Evaluation of the ecological niche model approach in spatial conservation prioritization
Source: PLoS One. 2019 Dec 20;14(12):e0226971. doi: 10.1371/journal.pone.0226971 (PMC6924678; doi:10.1371/journal.pone.0226971)
Supplement: S2 Figs — (PDF) [file pone.0226971.s004.pdf]

S2 Figs. Performance of the ecological niche model (ENM) approach using GAM.

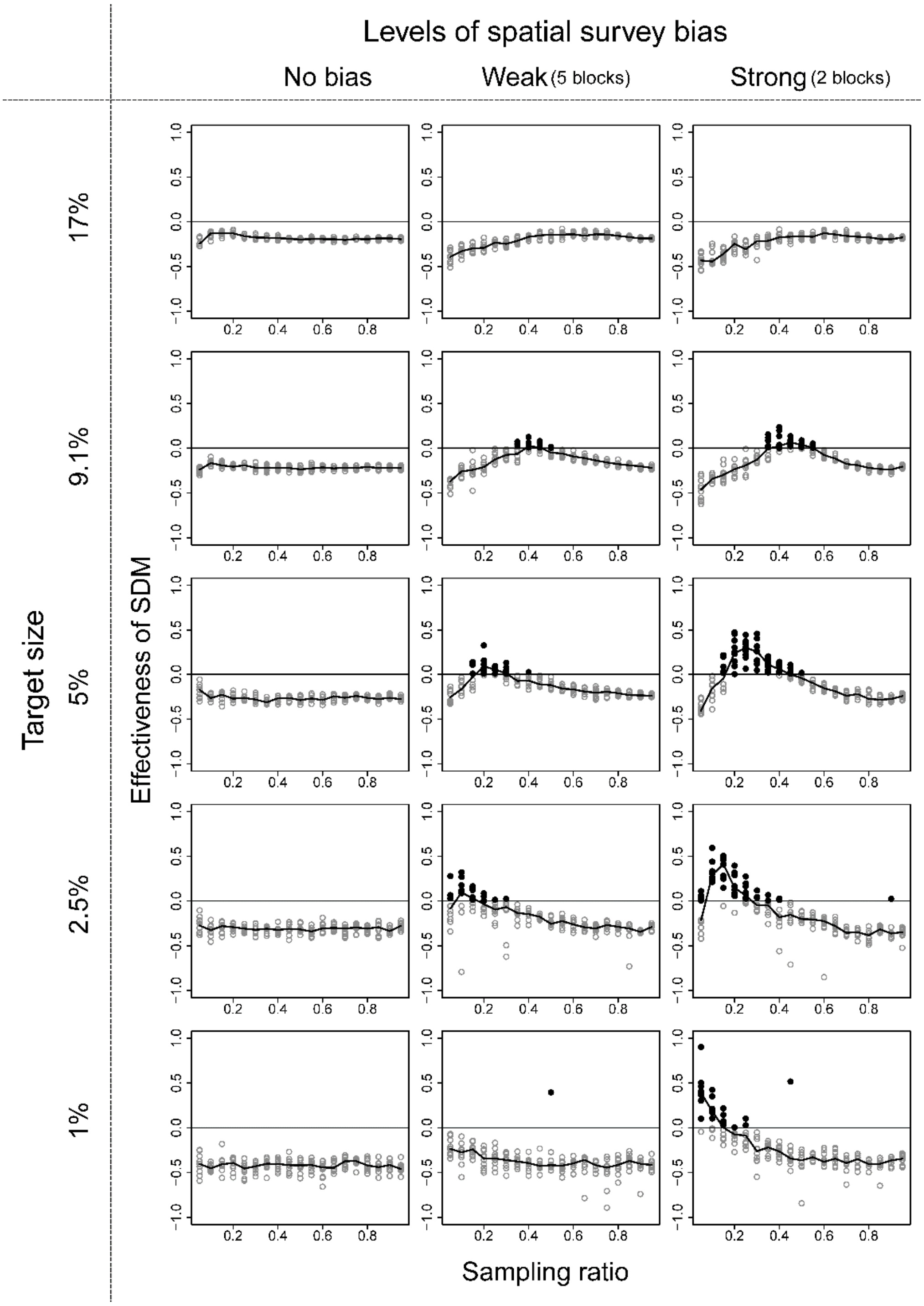

Fig A Performance of the ecological niche model (ENM) approach using GAM in the community structure type 1 illustrated in Figs 2a and b. Black filled circles are values when the SDM approach is beneficial, and gray open circles are the other case. Line chart shows median value in each condition.

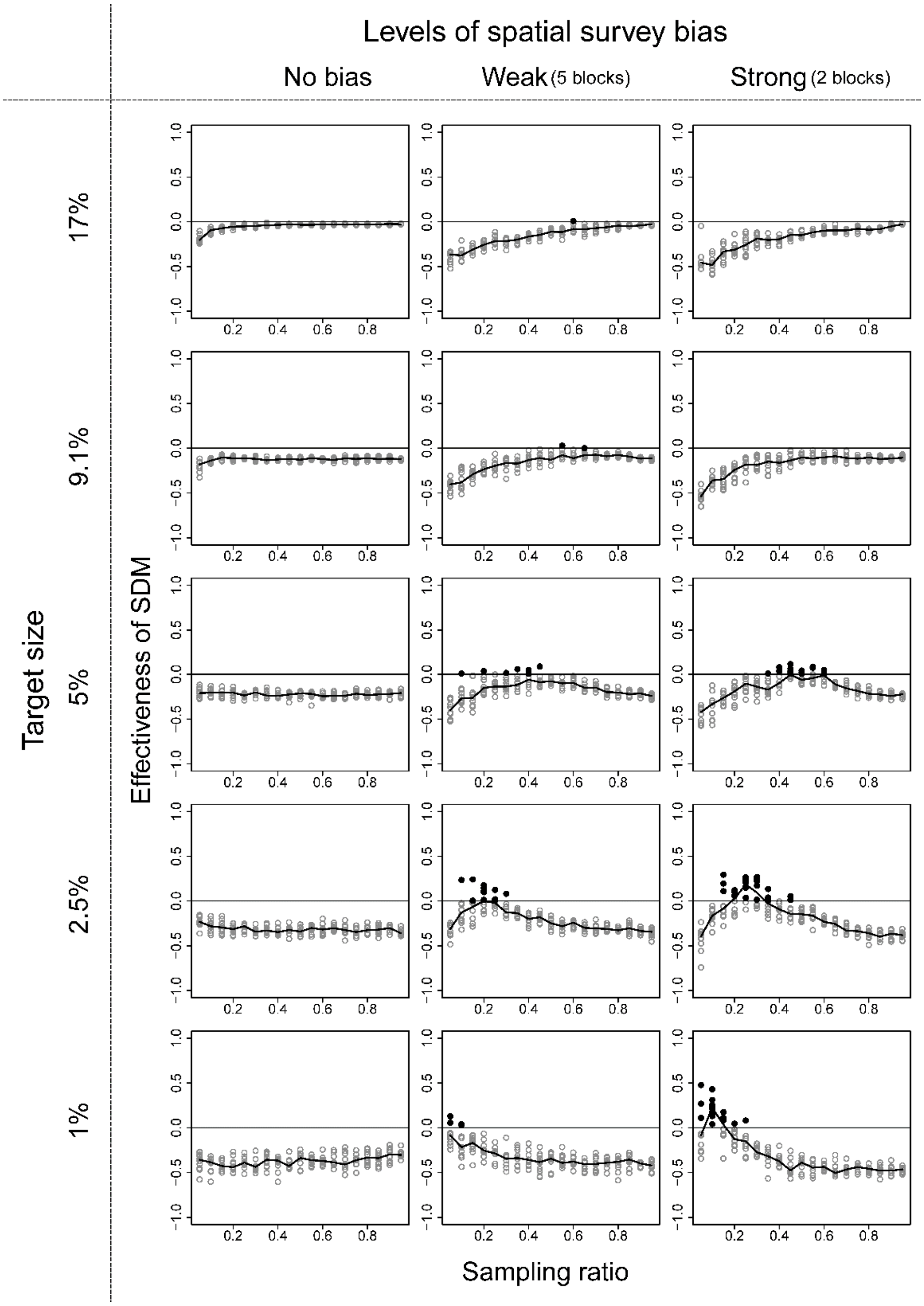

Fig B Performance of the ecological niche model (ENM) approach using GAM in the community structure type 2 illustrated in Figs 2c and d. See Fig A in S3 Figs for more details.

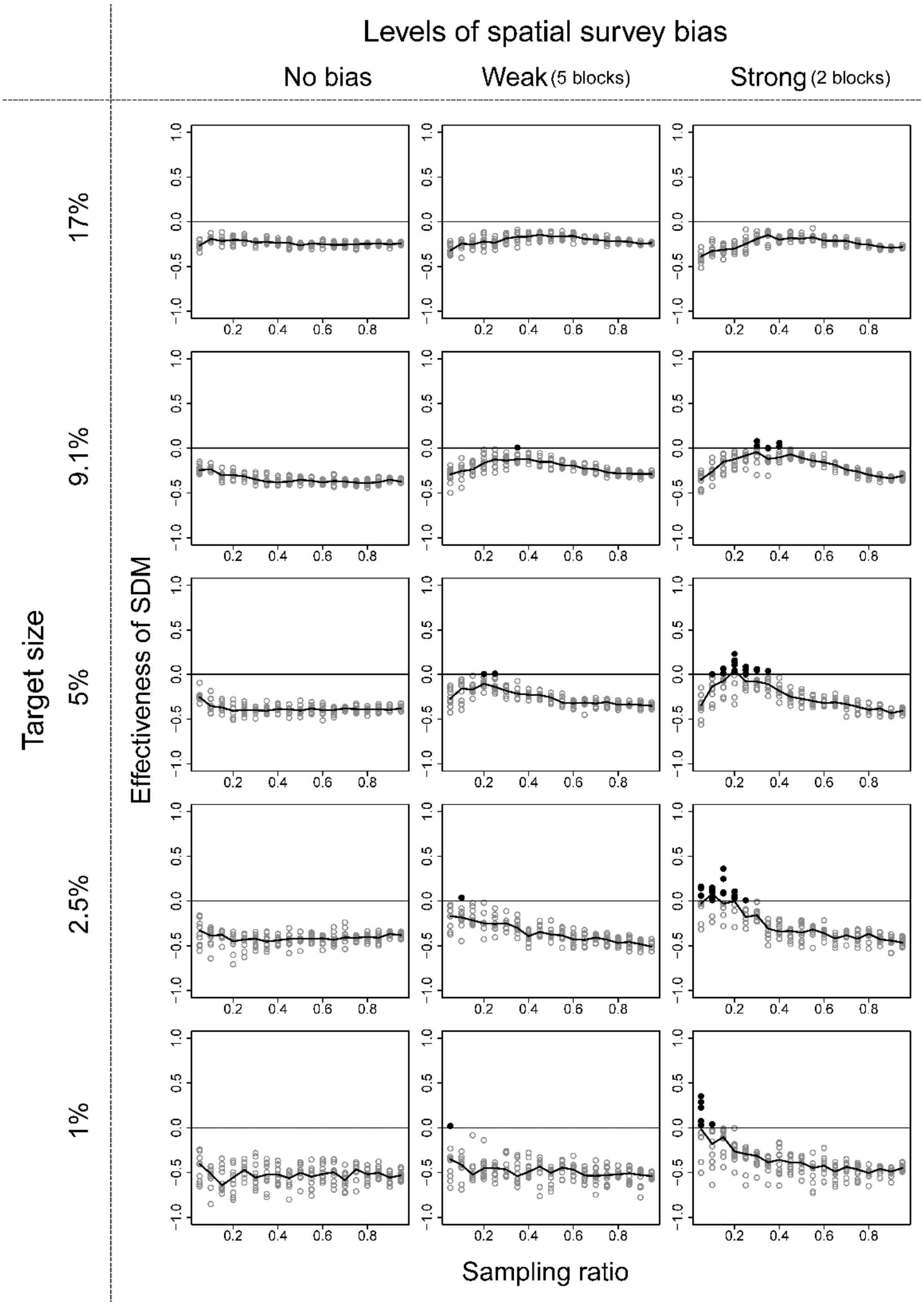

Fig C Performance of the ecological niche model (ENM) approach using GAM in the community structure type 3 illustrated in Figs 2e and f. See Fig A in S3 Figs for more details.
