# Supplementary figures and images for: Evaluation of the ecological niche model approach in spatial conservation prioritization
Source: PLoS One. 2019 Dec 20;14(12):e0226971. doi: 10.1371/journal.pone.0226971 (PMC6924678; doi:10.1371/journal.pone.0226971)

## GLM

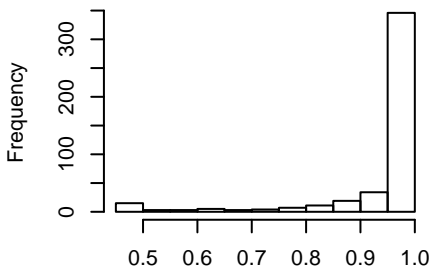

## GAM

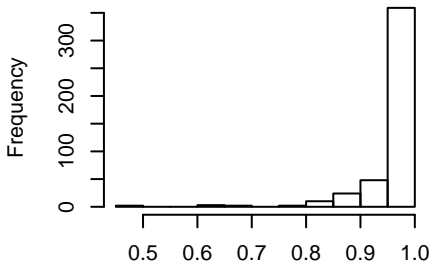

## Random Forest

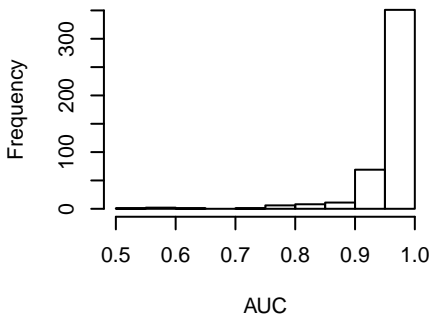

Supplement: S4 Figs — (PDF) [file pone.0226971.s006.pdf]
